# Decoding social decisions from movement kinematics

**DOI:** 10.1016/j.isci.2022.105550

**Published:** 2022-11-10

**Authors:** Giacomo Turri, Andrea Cavallo, Luca Romeo, Massimiliano Pontil, Alan Sanfey, Stefano Panzeri, Cristina Becchio

**Affiliations:** 1 Cognition, Motion and Neuroscience Laboratory, Center for Human Technologies, Istituto Italiano di Tecnologia, Genova, Italy; 2 Department of Psychology, University of Turin, Torino, Italy; 3 Department of Information Engineering, Università Politecnica delle Marche, Ancona, Italy; 4 Computational Statistics and Machine Learning Laboratory, Center for Human Technologies, Istituto Italiano di Tecnologia, Genova, Italy; 5 Department of Computer Science, University College London, London, UK; 6 Donders Institute for Brain, Cognition and Behavior, Radboud University, Nijmegen 6525 EN, the Netherlands; 7 Behavioral Science Institute, Radboud University, Nijmegen 6525 HR, the Netherlands; 8 Department of Excellence for Neural Information Processing, Center for Molecular Neurobiology (ZMNH), University Medical Center Hamburg-Eppendorf (UKE), Hamburg, Germany; 9 Neural Computational Laboratory, Center for Human Technologies, Istituto Italiano di Tecnologia, Genova, Italy; 10 Department of Neurology, University Medical Center Hamburg-Eppendorf (UKE), Hamburg, Germany

**Keywords:** Social interaction, social sciences, research methodology social sciences

## Abstract

Decisions, including social decisions, are ultimately expressed through actions. However, very little is known about the kinematics of social decisions, and whether movements might reveal important aspects of social decision-making. We addressed this question by developing a motor version of a widely used behavioral economic game - the Ultimatum Game - and using a multivariate kinematic decoding approach to map parameters of social decisions to the single-trial kinematics of individual responders. Using this approach, we demonstrated that movement contains predictive information about both the fairness of a proposed offer and the choice to either accept or reject that offer. This information is expressed in personalized kinematic patterns that are consistent within a given responder, but that varies from one responder to another. These results provide insights into the relationship between decision-making and sensorimotor control, as they suggest that hand kinematics can reveal hidden parameters of complex, social interactive, choice.

## Highlights

• How individuals move reveals their decisions in the Ultimatum Game
• Hand kinematics contains predictive information about both fairness and choice
• Choice and fairness information is expressed in idiosyncratic kinematic patterns

## Graphical abstract

**Figure:**
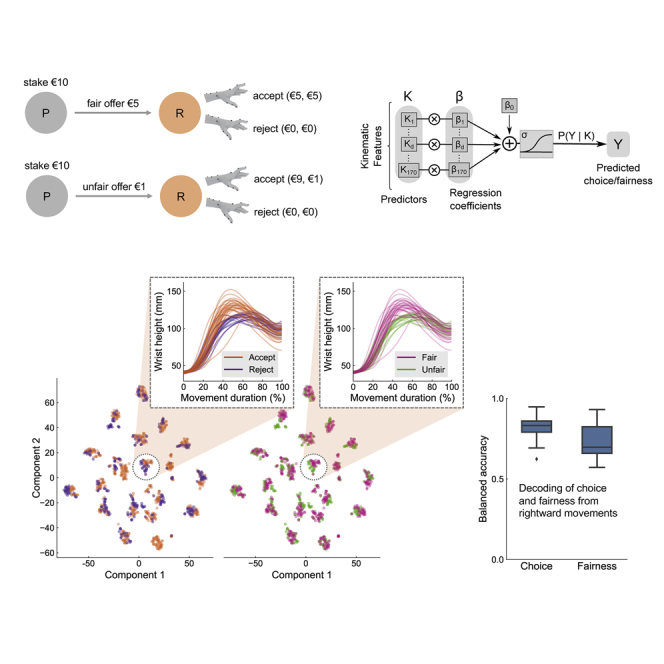

## Introduction

A convergence of modeling, behavioral, and neural data indicates that the way individuals move can provide important insights into cognitive states and ongoing decision processes.^1^^,^^2^^,^^3^^,^^4^^,^^5^^,^^6^ For example, in choice paradigms, the trajectory of reaching movements reveals not only the chosen option, reflected by the reaching direction but also the degree of confidence with which the choice is made, reflected by the hand-speed.^7^^,^^8^ Reaching kinematics can be used to infer intention,^9^^,^^10^ categorization dynamics,^11^ and reward associations.^12^ Moreover, specific events within reaching trajectories can be used to detect changes of mind^13^^,^^14^ and changes in confidence.^7^

These aforementioned studies examined individual choices, which typically involve clearly defined probabilities and outcomes. However, many of our most important decisions are made in the context of social interactions and are based on the concurrent decisions of others. These social decisions affect not only ourselves but also others and are therefore shaped by both self- and other-regarding motives.^15^^,^^16^^,^^17^ An example of this is when we decide whether or not to help another person—how we balance our aversion to unequal outcomes with economic self-interest.

Only a few studies have considered the possibility that reaching parameters may be useful in interpreting these kinds of social decisions. For example,^18^ found that in a two-person-social dilemma game, the trial-averaged trajectories were more curved when individuals defected than when they cooperated, suggesting that defection entailed more conflict. However, whether social decisions may be predicted from the kinematic parameters of individual reaching movements remains an unexplored question.

Here we designed a direct test of this hypothesis by developing a motor version of a widely used behavioral economic game, the Ultimatum Game.^16^ In this task, two players—a proposer and a responder—are given the opportunity to split a sum of money in a single interaction. The proposer makes an offer as to how the money should be split. The responder has the option to either accept or reject this offer. If the offer is accepted, the sum is divided as proposed. If it is rejected, neither player receives anything. Game theory predicts that a rational, self-interested, responder will accept any non-zero offer. However, experimental evidence contradicts this prediction. Responders accept fair offers close to the equal split but generally reject low offers, considering them unfair.^19^ Of course, punishing the proposer for a low offer is costly for the responder, and therefore the responder faces a conflict between the decision to either accept the low offer, and satisfy economic self-interest, or to reject it, based on inequity aversion,^20^ negative reciprocity,^21^ or reputational concerns.^22^

The decision to accept or reject is ultimately communicated through an action. For example, participants accept or reject the offer by pressing one of the two buttons.^23^^,^^24^ In a classical neuroeconomic setting, however, this action is considered merely a means of reporting the choice, and no study has yet examined the relationship between movement kinematics and decision parameters. Do accept and reject preferences influence the way individuals move toward the chosen option? Is the subjective assessment of the received offer reflected in the responders’ movements?

One difficulty in addressing these questions is the high variability of movement kinematics across repetitions of the same movement and across individuals - kinematics vary from one trial to another, and from one individual to another.^25^ A common approach is to average kinematics both over trials and individuals to reduce the effect of movement variability. However, because trial-to-trial and individual-to-individual variations often exceed decision-predictive variations, averaging can obscure how decisions map onto movement parameters. An alternative to averaging is to apply multivariate decoding methods as a tool for investigating information specified in movement kinematics at the single-subject, single-trial level.^6^^,^^10^^,^^26^^,^^27^^,^^28^ Here, we applied multivariate decoding to explore decision-predictive information encoded in the kinematics of individual responders in the one-shot Ultimatum Game. This approach enabled us to identify highly personalized patterns specifying predictive information about both the fairness of a received offer as well as the choice to either accept or reject that offer.

## Results

**Figure 1:**
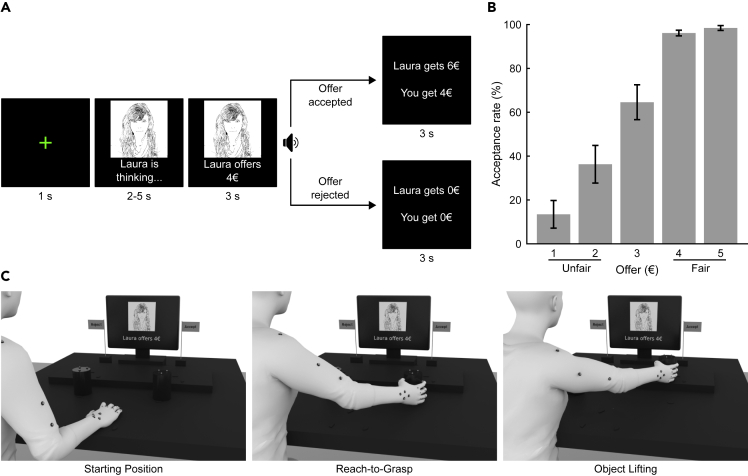
Trial design, experimental design, and behavioral results (A) Trial design of the Ultimatum Game task. (B) Average acceptance rates (±SEM) of the 20 responders. (C) Schematic of experimental design.

We used motion capture to track the arm kinematics of 20 participants while they played a motor version of the one-shot Ultimatum Game. Participants completed two sessions, always in the role of responder. They were told that in each session they would partner with 68 proposers recruited online and play a single iteration of the game with each proposer via a computer interface (Figure 1A). Offers from a pot of €10 were made according to a predetermined algorithm, which ensured that all participants received the same set of fair (€5, €4), mid-range (€3), and unfair (€2, €1) offers. On each trial, participants were instructed to respond by reaching out, grasping, and lifting one of the two cylinders labeled ‘accept’ and ‘reject’, located to the left/right of the body midline, respectively (Figure 1C). We reasoned that if the same object in the same location is grasped differently depending on a responder’s choice (accept versus reject), then variations in movement kinematics reflect the choice itself. By the same logic, if the same object, in the same location, is grasped differently depending on the fairness of the offer (fair versus unfair), then variations in movement kinematics reflect a response to the perceived fairness of the offer, independent of the subsequent choice. To ensure that any effects were not due to the direction of movement, we reversed the positions (left and right) of ‘accept’ and ‘reject’ cylinders across sessions.

### Acceptance rates

Acceptance rates, computed as the number of accepted offers divided by the number of proposed offers across each offer level, were similar to those previously reported in the Ultimatum Game literature.^17^ Participants accepted almost all fair offers (€5: 98.4 ± 1.1%, €4: 96.13 ± 1.3%, mean ± SEM), with decreasing acceptance rates as the offers became less fair (€3: 64.6 ± 7.9%; Figure 1B). Unfair offers (€2, €1) were accepted only 36.3 ± 8.6% and 13.4 ± 6.3% of the time, respectively.

### Clustered single-responder representations

**Figure 2:**
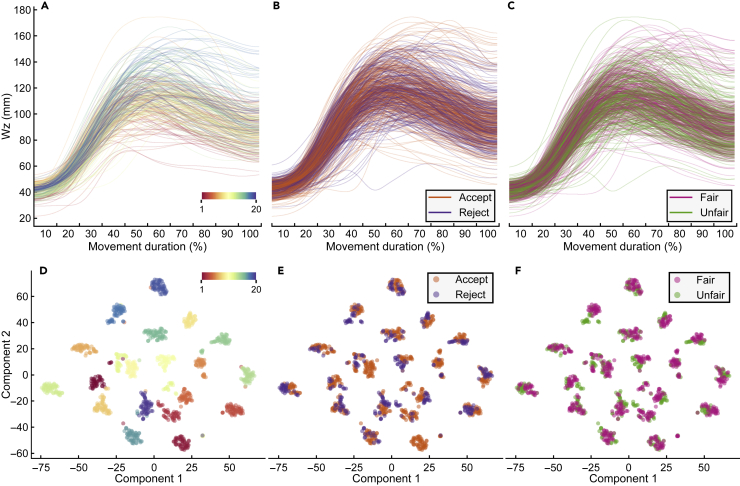
Single-trial movement kinematics (A–C) Representative kinematic traces of wrist height (W_z_) graphed by responder’s identity (A), choice (B), and fairness (C). Each line is an individual reach-to-grasp movement. (D–F) Application of t-SNE to movement traces. Each point represents an individual reach-to-grasp movement embedded into a two-dimensional space using t-SNE. Points are color-coded based on the responder’s identity (D), choice (E), and fairness (F).

Kinematic traces revealed large variability across trials and individuals. Figures 2A–2C show representative movement traces toward right targets clustered by responder’s identity, choice, and fairness. Each line is a reach-to-grasp action. To examine whether there was structure in this behavioral variability, as an exploratory step, we applied a non-linear dimensionality reduction technique, namely the t-distributed stochastic neighbor embedding (t-SNE)^29^ (Figures 2D–2F), to reduce the dimensionality of kinematic data to two dimensions. The proximity of traces in this reduced space reflects the similarity of traces in the high-dimensional kinematic space. t-SNE revealed a reliable segmentation of the ∼1000 traces into 20 isolated clusters. Color coding traces based on responders’ identities revealed that each cluster almost perfectly identified an individual responder (Figure 2D). Within each cluster, traces showed further separation between accepted and rejected offers (Figure 2E), as well as between fair and unfair offers (Figure 2F). This suggests that social decision parameters are expressed in individualized motor patterns.

### Decoding choice and fairness from movement kinematics of individual responders

To determine the relationship between social decision parameters and trial-to-trial variations in each responder’s kinematics, we trained, separately for each responder, a logistic regression classifier to predict the responder’s upcoming choice (accept versus reject) based on the unfolding of movement parameters during individual trials. By the same logic, we trained a logistic regression classifier to predict, for each responder, the fairness of the proposed offer (fair versus unfair). The training set comprised, for each responder, fair (€4, €5) and unfair (€1, €2) offers. Logistic regression classifiers find a set of linear weights on kinematic features that maximize the cross-validated probability of correctly decoding the decision parameter for the individual responder. To ensure that these weights reflected true kinematic profiles (and not mixtures of movements toward left and right targets), we trained separate logistic regression classifiers for the left and right targets. We compared responder-specific logistic regression models with a set of alternative responder-specific classifiers of varying form and complexity. We verified that logistic regression classifiers performed better than or comparable to the alternative classifiers (see Figure S2; Table S2).

**Figure 3:**
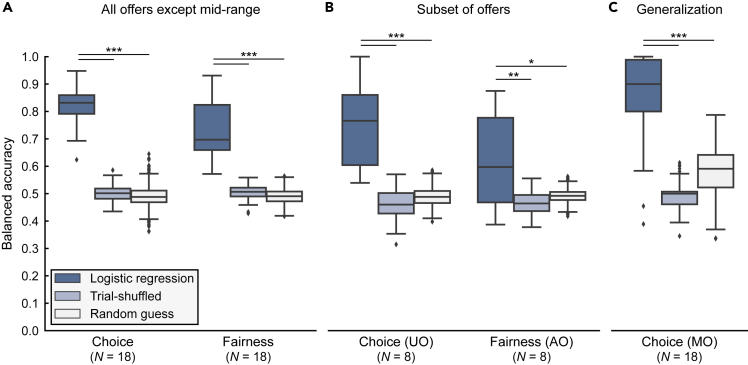
Performance of logistic regression classifiers trained with rightward movements (A) Boxplots of balanced prediction accuracies of responder-specific logistic regression classifiers trained on rightward movements to predict choice and fairness. Prediction accuracies were significantly higher for actual data than for trial-shuffled data and random guesses. (B) Boxplots of balanced prediction accuracies for choice classification on unfair trials only (UO), and fairness classification on accept trials only (AO). (C) Boxplot of balanced prediction accuracies for choice classification on mid-range offers (MO). ∗ indicates p < 0.05, ∗∗ indicates p < 0.01, and ∗∗∗ indicates p < 0.001. *N* indicates the number of responders included in each analysis.

Figure 3A shows the balanced prediction accuracies of individual responder classifiers trained on rightward movements. For both choice and fairness classifications, prediction accuracies were significantly higher than those expected for trial-shuffled data (in which the association between kinematic data and choice/fairness labels had been removed by shuffling) or random guesses (Figure 3A and Table S1). The balanced prediction accuracies of individual responder classifiers trained on leftward movements were qualitatively similar (Figure S3 and Table S1).

The above results were obtained by training and testing separate logistic regression classifiers for each responder. In a control analysis, to test the individuality of motor patterns, we trained a logistic regression classifier using data from all but one responder and then tested them on the left-out responder. If patterns are idiosyncratic, we would not expect classifiers to generalize to the unseen responder. In line with this prediction, classifiers trained with this leave-one-subject-out cross-validation scheme showed chance performance (Figure S4 and Table S1), indicating that the relationship between kinematics and social decision parameters learned in one responder was not generalizable to other responders.

### Disentangling choice and fairness information

The above results suggest that both the fairness of a proposed offer as well as the decision to accept or reject that offer can be predicted from the single-trial kinematics of individual responders. However, given the dependence between fairness and choice—the probability of accepting a fair offer being three times the probability of accepting an unfair offer across trials—the above analysis cannot rule out the possibility that choice-related variations contribute to ‘fairness’ predictions, and by the same logic, fairness-related variations in movement kinematics contribute to ‘choice’ predictions.

To decouple the contribution of choice-predictive and fairness-predictive information, we examined the possibility of predicting choice from the kinematics of unfair trials only, and conversely, the possibility of predicting fairness from the kinematics of accepted trials only (because of the few rejected fair trials, predicting choice from fair trials only and predicting fairness from rejected trials only was not possible). Conditioning the prediction of one class (e.g., choice) on a particular value of the other (e.g., fairness) discounts the effect of the latter on the prediction of the former. As shown in Figure 3B, for both classes, the conditional predictions were still consistently superior to those of both trial-shuffled data and random guesses (Table S1). Taken together, these analyses suggest that movement kinematics contain information about both choice and fairness.

### Kinematic choice patterns trained on fair and unfair offers generalize to mid-range offers

The above-described responder-specific choice predictions were trained and tested on fair (€5, €4) and unfair offers (€2, €1), excluding mid-range offers (€3). As a way of assessing the generalizability of kinematic patterns that discriminate between accept and reject choices across offer levels, we tested the ability of responder-specific choice classifiers, trained on fair (€5, €4) and unfair offers (€2, €1) of a given responder, to predict the responder’s choice for mid-range offers (€3), not used for training. As shown in Figure 3C, the median balanced prediction accuracy across responders was again close to 90% (Table S1). We, therefore, conclude that, within an individual responder, single-trial kinematics reflect choice information above and beyond the monetary value of the proposed offer.

### Kinematic codes for fairness and choice

**Figure 4:**
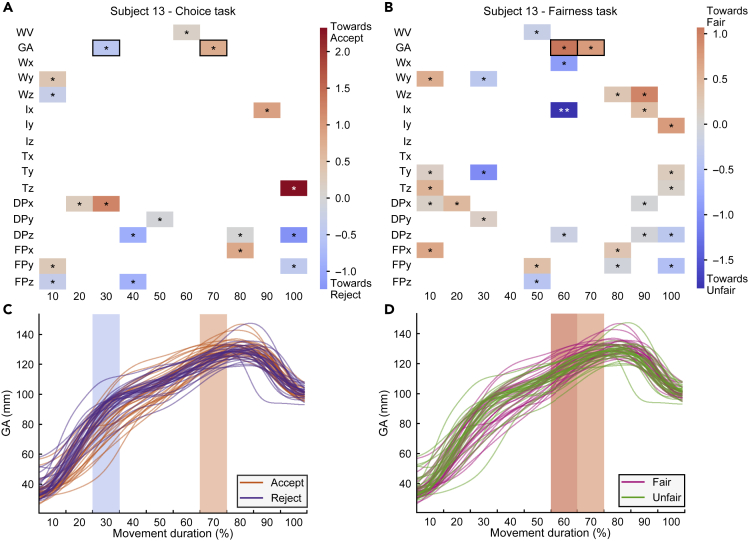
Encoding of choice and fairness in the kinematics of an individual responder (A and B) Average logistic regression weights for choice (A) and fairness (B) classifications of rightward movements of an individual responder. (C and D) Time course of grip aperture (GA) of the same responder graphed by choice (C) and fairness (D). Each line is an individual reach-to-grasp movement. Time bins corresponding to significant positive and negative weights are highlighted. ∗ indicates p < 0.05, ∗∗ indicates p < 0.01, and ∗∗∗ indicates p < 0.001.

Having validated the capability of logistic classifiers to predict choice and fairness, we next used them to investigate how information about social decision parameters is specified in the kinematics of individual responders. Figure 4 visualizes the contribution (weight) over time of each kinematic feature for both choice predictions (Figure 4A) and fairness predictions (Figure 4B) for the rightward movements of an individual responder. A positive (negative) logistic regression weight is assigned to a feature that, over trials, is distributed with higher (lower) values for accept compared to reject choices, and for fair compared to unfair offers. For example, at 30% of movement duration, grip aperture (GA) is larger for reject choices and thus is assigned a negative value (Figure 4C). This pattern reverses around 70% movement duration when GA is assigned a positive weight. Similarly, GA is larger for fair compared to unfair trials around the time of the maximum hand aperture and is thus assigned a positive weight at 60 and 70% of movement duration for fairness predictions (Figure 4D).

**Figure 5:**
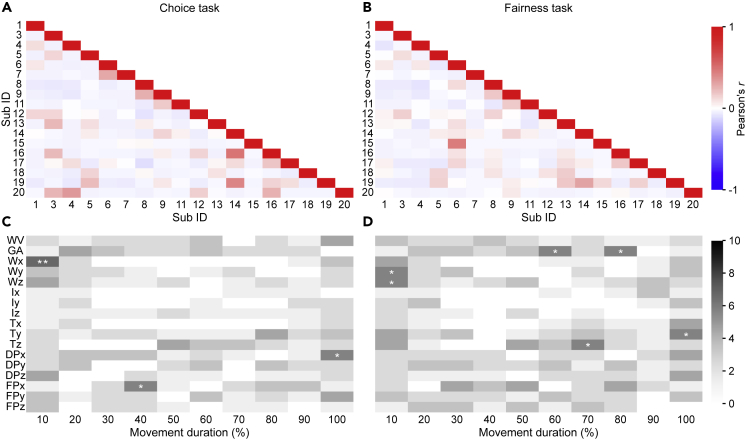
Overlap of choice and fairness weights across responders of rightward movements (A and B) Pearson correlation of the average logistic regression weights between each pair of responders for rightward movements for choice (A) and fairness (B) classification. (C and D) Number of responders, for each feature, for which the feature was statistically significant for choice (C) and fairness (D) classification. ∗ indicates p < 0.05, ∗∗ indicates p < 0.01, and ∗∗∗ indicates p < 0.001.

To quantify the degree of (dis-)similarity of kinematic patterns across responders, we computed, separately for choice and fairness, the correlation between the weights of each responder and those of all other responders, separately for rightward movements (Figures 5A and 5B) and leftward movements (Figures S5A and S5B). As expected, regression weights correlated weakly across responders, corroborating the idea that the encoding of social decision parameters is idiosyncratic. Nonetheless, it remains possible that across responders some features were used more than others. As an attempt to identify features used more often across responders, we plotted, for each feature, the number of responders for which the feature carried significant choice or fairness information (Figures 5C and 5D). This revealed a widely distributed use of features to encode information. Next, we individuated those features (marked with stars in Figures 5C and 5D) that were used for encoding by a number of responders higher than expected if encoding was distributed randomly across the kinematic space (Table S3). As shown in Figures 5C and 5D, this analysis produced sparse maps, with few common features expressed, mainly observed at 10 and 100% of movement duration (Table S3). This is expected because the start position (hand resting on the table) and the final position (hand on the cylinder) are relatively constrained, and inter-individual variability is lower at these time epochs (see Figure S6). Individualized patterns are thus more likely to overlap at these epochs.^25^ The most used feature (W_x_ at 10% of movement duration) was employed by 9 out of 18 participants. Similar results were obtained when considering leftward movements (Figures S5C and S5D). Viewed collectively, these results suggest that movement traces encode choice and fairness information and that this information is expressed in highly personalized kinematic patterns, with only a few features common to sub-populations of responders.

## Discussion

Social decisions have been almost exclusively studied in disembodied economic settings, in which the action component is somewhat of an afterthought, reduced to a stereotyped button press.^3^ In the real world, however, social decisions are embodied into actions that require forward planning (i.e., what am I going to do next),^6^ that have associated costs,^4^^,^^18^ and that can be observed by others.^3^ This raises the question of whether, and to what extent, social decision parameters might be reflected in action parameters.

To answer this question, we used a multivariate single-subject, single-trial approach to decode key social decision parameters from the kinematics of responders playing a novel motor version of the Ultimatum Game. In this game, the responder faces a conflict between the decision to accept any non-zero offer, and thus maximize self-economic benefit, and the decision to reject non-equitable proposals, and thus punish unfairness.^19^ Our approach revealed that movement contains predictive information about both the fairness of a proposed offer and the decision to either accept or reject that offer. These results suggest that how individuals move toward a choice option reflects social decision parameters and provides an ongoing readout of social decision dynamics.

### Individuality in motor coding of social decisions

Each responder embodied a parametrization of choice and fairness information that was both consistent within a given responder and varied from one responder to another. This observation adds to the growing body of evidence documenting the individuality of motor solutions.^25^ Consistent with the suggestions that individual variability is high where the effect of motor output is low,^25^ we found that individual differences were most expressed while the hand was mid-air *en route* to the object.

It is tempting to speculate that above and beyond biomechanics, these differences may reflect differences in how the brain computes social decision parameters. Evidence from brain imaging studies using the Ultimatum Game^30^ indicates that both choice and fairness information are represented in a distributed brain network that prominently includes the anterior insula, anterior cingulate cortex, dorsolateral and dorsomedial prefrontal cortex, supplementary motor area, cerebellum, and putamen. Future studies could examine whether and to what extent variations in fMRI signal within this network covary with movement kinematics.

Leveraging inter-responder differences in activation, covariance analyses between fMRI and kinematic data could then be conducted to identify the motion signatures of different decision strategies. Using fMRI while participants played the Trust Game, a task closely related to the Ultimatum Game, van Baar et al.^31^ found markedly different individual neural substrates for different decision strategies, even under conditions where the two strategies produce the same behavioral output. For example, inequity-averse subjects, motivated by a principled egalitarian rule, shared a distinctive activity pattern in ventromedial prefrontal cortex, dorsal anterior cingulate cortex, supplementary motor area, and bilateral superior occipital cortex. In contrast, guilt-averse subjects, this time motivated by an aversion to harming others, shared a particular activity pattern encompassing bilateral anterior insula, bilateral putamen, dorsomedial prefrontal cortex, and left dorsolateral prefrontal cortex. This demonstrates the utility of considering individual differences in motivations across the social choice. Future empirical and modeling studies could usefully examine how kinematics covaries with activity in networks of regions associated with different individual strategies.

Finally, having learned how different strategies are reflected in differences in movement kinematics, future studies could reverse the inference and use kinematics to infer which strategy responders are using and when they are switching to another strategy. Individual decision strategies tend to be consistent across different contexts.^32^ However, individuals may apply strategic variability and switch between strategies. Movement kinematics may provide a means to discriminate between strategies and detect changes in strategy.

### Readout of the decision process during social interactions

In authentic social situations, people interact with others. An implication of our findings is that movement parameters expressing social decisions can be potentially exploited by other people. In support of this notion, human perceivers can use subtle differences in movement kinematics to predict intention,^9^^,^^10^ discern deception,^33^ and even infer the value of a poker hand from subtle variations in movement kinematics.^34^ This suggests that human perceivers are sensitive to information encoded in movement kinematics.^6^ However, further work is needed to explore the intriguing possibility of whether this sensitivity could extend to understanding motivational strategy and choice information from movement kinematics.

One potential challenge here is related to inter-responder variability of kinematic traces. Under these varying conditions, it might be difficult, if not impossible, for human perceivers to identify common features diagnostic of choice across responders. A strategy to deal with this variability might be to combine different sources of information, for example, kinematics and gaze behavior.^35^^,^^36^^,^^37^ In sequential-presentation paradigms, final fixations on alternatives have been shown to be predictive of the subsequent other-regarding choices.^37^ Future studies could test whether human perceivers are able to integrate information transmitted by gaze and hand behavior to predict social decisions.

### Linking social decisions and sensorimotor control

By tracking fairness and choice in reach-to-grasp kinematics, we examined the possibility that parameters of social decisions influence sensorimotor control. Our single-subject, single-trial findings document a specific influence of fairness and choice on the kinematics of reach-to-grasp movements, above and beyond a target motor representation. These results provide a critical addition to the literature linking decision-making and sensorimotor control by suggesting that hand kinematics can reveal hidden parameters not only of individual decisions but also of more complex social decisions.

### Limitations of the study

Here, we emphasize the individuality in the motor coding of social decisions. However, our results do not exclude that individual motor signatures cluster into a limited number of motor phenotypes. Much larger sample sizes would be needed to test this hypothesis using unsupervised clustering procedures.^32^

Further investigation is also required to establish the consistency of individual patterns across different strategic settings. Our results show that individual choice patterns for fair/unfair offers generalize to mid-range offers. If individuals exhibit similar kinematic patterns in different experimental settings, this would provide a more robust empirical case for the idea of an individual choice (and fairness) signature.

## STAR★Methods

### Key resources table

**Table:** 

REAGENT or RESOURCE	SOURCE	IDENTIFIER
**Deposited data**		

Data supporting main findings	This paper	https://data.mendeley.com/datasets/jrtjw734r3

**Software and algorithms**		

Vicon Nexus	Vicon Motion Systems Ltd UK	https://www.vicon.com/products/software/nexus; RRID:SCR_015001
MATLAB	MathWorks Inc	https://it.mathworks.com/products/matlab.html; RRID:SCR_001622
Python	Python software foundation	http://www.python.org/; RRID:SCR_008394
PyTorch	Meta AI	https://pytorch.org/; RRID:SCR_018536
R Project for Statistical Computing	The R Foundation	http://www.r-project.org/; RRID:SCR_001905
E-Prime	Psychology Software Tools	http://www.pstnet.com/eprime.cfm; RRID:SCR_009567

### Resource availability

#### Lead contact

Further information and requests for resources should be directed and will be fulfilled by the lead contact, Cristina Becchio (c.becchio@uke.de).

#### Materials availability

This study did not generate new unique reagents or materials.

### Experimental model and subject details

Based on previous work using logistic regression to decode intention from movement kinematics (e.g.,^10^), we aimed at testing 20 participants. Twenty-one participants completed the task. One participant was excluded from the data analysis because of extremely low acceptance rates of fair offers (acceptance rates were about 3.6 standard deviations below the group mean for both offers of 4 and €5). The remaining 20 participants (10 females; mean age 23; range 20-27) were right-handed with normal or corrected-to-normal vision. None of the participants reported neurological or psychiatric disorders. Written informed consent was obtained from each participant. The research was approved by a local ethical committee (ASL 3 Genovese) and was carried out in accordance with the principles of the revised Helsinki Declaration (World Medical Association General Assembly, 2008). All participants received monetary compensation proportional to the amount of money gained during the experiment (for details, see later in discussion). The dataset was collected before any analysis began, and no data was added subsequent to the beginning of analysis.

### Method details

#### Task

Participants played a motor version of the Ultimatum Game as a one-shot game. The Ultimatum Game can also be played with the same two partners interacting repeatedly, see for example.^38^ However, under these circumstances, the game morphs into a reputation game,^39^ changing both the optimal and actual game strategies. We used the one-shot Ultimatum Game because this version minimizes strategic motives and is thus best suited for studying fairness responses.^16^ Participants completed two sessions, always in the role of responder. They were told that in each session they would play each iteration of the game in real-time with a proposer selected from a pool of 68 different players from the Istituto Italiano di Tecnologia, with whom they would connect via a computer interface. To minimize the use of strategic considerations, they were informed that the same pool of players would participate in both sessions (so that each responder would receive two offers from each proposer) but that proposers would not be informed of responder decisions until the end of the game. The offer could be 1, 2, 3, 4, or 5 out of €10. Participants were informed that if they accepted the offer, the money would be split as proposed; if they rejected the offer, neither player would receive anything. Unbeknownst to participants, the responder was, in fact, computer-simulated, and all participants received the same set of offers.

#### Apparatus

Each participant sat on a height-adjustable chair, with their right hand and wrist resting on a table. The hand, wrist, and right forearm were oriented on the parasagittal plane passing through the shoulder, and the right hand was in a semi-prone position, with the tips of the thumb and index finger on a tape-marked point, placed on the working space. The workspace (width = 100 cm; length = 110 cm) was covered with black fabric. Two upright cylinders (height = 11 cm; diameter = 7.5 cm; weight = 104 g) were placed in front of the hand position at a comfortable reaching distance (44 cm from the table edge to object), 18 cm to the left and right from body midline. The cylinders were labeled ‘accept’ and ‘reject’. The proposed offer was displayed on each trial on a screen placed on the table together with the silhouette of the randomly selected proposer (see Figure 1C for a schematic representation of the experimental setup).

#### Procedures

Each trial started with a green fixation cross for 1 s presented at the center of the screen, followed by a silhouette of the proposer with the message ‘[Name of proposer] is thinking … ’ (e.g., ‘Laura is thinking … ’). This screen could last 2, 3, 4, or 5 s. The offer then appeared (e.g., ‘Laura’s offer is €4’), remaining visible for 3 s. Participants were instructed to make their choice within this time window by reaching out, grasping, and moving one of the two cylinders to a target platform (height = 3 cm; length = 27 cm; width = 50 cm). Two marked locations indicated where the cylinder should be placed. After placing the object, participants returned their hands to the starting position. After a tone, the money earned by each participant was displayed for 2 s (e.g., if the participant accepted the offer: ‘Laura gets €6, you get €4’, otherwise, if the participant rejected the offer: ‘Laura gets €0, you get €0’). Finally, a red fixation cross instructed participants to return, using their left hand, the cylinder to the home position. The trial design is depicted in Figure 1A.

As part of the cover story, participants were told that the selection of a proposer required at least 10 players to be connected at the beginning of the trial. If less than 10 players were connected, the computer would automatically generate the instruction to grasp one of the two cylinders (control trials). Each participant completed two sessions, separated by a short break. Each experimental session comprised 80 trials: 68 Ultimatum Game trials and 12 control trials (6 rightward, 6 leftward). In Ultimatum Game trials, each participant saw 12 €1 offers, 12 €2 offers, 12 €3 offers, 16 €4 offers, and 16 €5 offers. The number of offers was chosen based on previous studies^40^ to mimic the offer pattern of a human proposer. Participants were informed that the financial compensation would be proportional to the money gained during the experiment. We debriefed participants at the end of the experiments. Post-experimental interviews confirmed that participants were unaware of the purpose of the study and had believed the cover story. One participant (participant 8) expressed the doubt that proposers were not real players. We verified that excluding the participant did not affect any of the results.

The order of trials was fully randomized across participants. The ‘accept’ and ‘reject’ labels assigned to cylinders were counterbalanced across sessions. The silhouette was female (male) in half of the trials (34 Ultimatum Game trials). Using logistic mixed effects models, we verified that neither the gender of the silhouette (proposer) nor that of the responder had any effect on the decision to accept or reject the offer (Table S6). The E-Prime software (v.2.0.10.242) was used for the randomization of the trials and the synchronization with the kinematic acquisition. The experiment lasted for a total of 70 min.

#### Kinematic data acquisition

We recorded movement kinematics using a near-infrared motion capture system with eight cameras (acquisition frequency = 100 Hz; Motion Capture Vicon system). Cameras were positioned in a semicircle at about 1.5 m from the participant’s location. Each participant was outfitted with 30 retro-reflective markers (Ø = 4 mm) placed on the dorsal surface of the wrist (*wrist*) and hand (*palm*), radial and ulnar region of the wrist (*radio* and *ulna*), trapezoid bone of the thumb (*thu0*), tip, interphalangeal and metacarpophalangeal joints of the thumb (*thu3*, *thu2*, and *thu1* respectively), index (*ind3*, *ind2*, and *ind1*), middle (*mid3*, *mid2*, and *mid1*), ring (*rin3*, *rin2*, and *rin1*) and little finger (*lit3*, *lit2*, and *lit1*), lateral face of the elbow and arm, acromial process of right and left shoulder, sternal fork, xiphoid process of the sternum, and head (two frontal and two posterior, right and left). Three markers were also placed on the top of each cylinder. See Figure S1A for a layout of marker placement.

#### Kinematic data preprocessing and computation of kinematic variables

Each trial was individually inspected for correct marker identification and then run through a low-pass Butterworth filter with a 6Hz cutoff. Kinematic variables were chosen to provide a complete description of arm and hand kinematics during reaching and grasping. Specifically, we used custom software (Matlab; MathWorks, Natick, MA) to compute two sets of kinematic variables of interest: F_global_ variables and F_local_ variables. F_global_ variables, expressed with respect to the global frame of reference (the frame of reference of the motion capture system), included the following variables:

• wrist velocity, defined as the module of the three-dimensional velocity vector of the *radio* marker (in mm/s);
• x-, y-, and z-wrist, defined as the x-, y-, and z-component of the *radio* marker (in mm);
• grip aperture, defined as the Euclidean distance between the markers that were placed on the tips of the thumb (*thu3*) and the index finger (*ind3*; in mm); These variables served to characterize the arm kinematics. To characterize hand joint movements, we computed a second set of variables expressed with respect to a local frame of reference centered on the hand (i.e., F_local_). Within F_local_, we computed the following variables:
• x-, y-, and z-index, defined as the x-, y-, and z-component of the *ind3* marker (in mm);
• x-, y-, and z-thumb, defined as the x-, y-, and z-component of the *thu3* marker (in mm);
• x-, y-, and z-finger plane, defined as the x-, y-, and z-components of the thumb-index plane (defined by the markers *thu3*, *ind3*, and *ind1*). These components provide information about the abduction/adduction movement of the thumb and index finger irrespective of the effects of wrist rotation and of finger flexion/extension (Figure S1A);
• x-, y-, and z-dorsum plane, defined as the x-, y-, and z-components of the radius-phalanx plane projection (defined by the markers *ind1*, *lit3*, and *wrist*). These components provide information about the abduction, adduction, and rotation of the hand dorsum irrespective of the effects of wrist rotation (Figure S1A).

We have previously shown that these two sets of variables can be used to capture subtle differences between kinematics associated with different internal states.^9^^,^^10^^,^^41^^,^^42^^,^^43^ All variables were calculated only considering the reach-to-grasp phase of the movement, from reach onset (the first time at which the wrist velocity crossed a 20 mm/s threshold) to reach offset (the time at which the wrist velocity dropped below a 20 mm/s threshold). Having verified that movement duration did not vary as a function of choice or fairness (p > 0.1 for both parameters and movement directions), kinematic variables were normalized into a percentage of movement duration and analyzed as a continuous series of 10 epochs (0%–10%, 10%–20%, …, 90–100%), resulting into 170 kinematic features.

### Quantification and statistical analysis

#### Single-trial kinematic vector

We summarized the kinematics of each reach (i.e., trial) as a vector in the 170-dimensional kinematic space spanning the 17 kinematic variables over 10-time epochs.

#### t-SNE

For visualization, we mapped the 170-dimensional single-trial vector of each trial onto a low-dimensional subspace with t-distributed stochastic neighbor embedding (t-SNE) (Figure 2). The perplexity parameter was set to 15 (similar results were obtained with a wide variety of parameters). Traces were then color-coded based on the identity of responders (Figure 2D), the choice to accept and reject the proposed offer (Figure 2E), and the fairness of the proposed offer (Figure 2F). We chose t-SNE because of its ability to work even in the presence of non-linear relationships between features and outliers.^29^

#### Decoding of choice and fairness with responder-specific logistic regression

We used logistic regression, similar to,^10^ to classify choice (accept versus reject) and fairness (fair versus unfair) from the multivariate single-trial kinematic vector, defined as above.

The logistic regression choice classifier estimated the probability that a reach expressed the decision to accept the proposed offer as a sigmoid transformation of the kinematic vector in that trial. The equation of the logistic regression model was as follows:

$$P\left(y=1|K\right)=\sigma \left(\beta_{0}+\beta^{T}K\right)=\frac{1}{1+e\left(\beta^{T}K+\beta_{0}\right)}$$

P(y=0|K)=1−P(y=1|K)

where *σ* is the sigmoid function, *K* is the kinematic vector, *y* is the binary response variable (*y = 1*, if the offer is accepted; *y = 0*, if the offer is rejected), *β* is the vector of the regression coefficients (weights), and *β*_*0*_ is a bias term.

Similarly, the logistic regression fairness classifier estimated the probability that a reach responded to a fair offer as a sigmoid transformation of the kinematic vector in that trial. The regression model equation was the same as that of the choice regression model, except that the regression was performed using a binary response variable for fairness (*y = 1*, if the proposed offer is fair; *y = 0*, if the proposed offer is unfair). See Figure S1B for a block diagram of the model.

Both choice and fairness regression classifiers were trained separately for each responder. To be included in the analysis, each responder had to contribute a minimum of four trials in each class in each classification task. The number of trials available for individual responders is reported in Tables S4 and S5.

#### Training of logistic regression models

To avoid penalizing predictors with larger value ranges, we z-scored the single-trial kinematic vectors within each responder. With the response variable encoded as 0 or 1, a penalized version of the logistic regression classifier was trained by minimizing the negative binomial log likelihood *L*_*LR*_ with an elastic net regularization^44^ defined as:

$$\underset{\beta ,\beta_{0}}{\min\;}L_{LR}\left(\beta ,\beta_{0}\right)=\min_{\beta ,\beta_{0}}-\left[\frac{1}{n}\sum_{i}^{n}w_{i}\left(\beta^{T}K_{i}+\beta_{0}\right)-\log\left(1+e^{\beta^{T}K_{i}+\beta_{0}}\right)\right]$$

$$+\lambda \left[\frac{\left(1-\alpha \right)\|\beta {\|}_{2}^{2}}{2}+\alpha {\left\|\beta \right\|}_{1}\right]$$

where *n* is the total number of trials, λ is the regularization parameter, *α* is a value between 0 and 1 weighing the relative contribution of the L1 and L2 penalty, and *w*_*i*_ is the rescaling weight assigned to the *i-th* trial that is inversely proportional to class frequencies of training data.

The hyperparameter λ was tuned using a nested leave-one-trial-out cross-validation (LOTO-CV) procedure. We used all data available for each responder. An evaluation of how model performance scales with the amount of data is provided in Figure S7, in which we repeated the responder-specific LR classifications as described here but using only 50% or 75% of the data available for each responder, rather than the full dataset. A grid search procedure was used to find the best λ value within the range logspace(1, −3, 5). To obtain sparser, and thus more interpretable solutions, *α* was fixed at 0.95. We used *α* = 0.95 because using this value, as shown in,^44^ provides numerical stability. To investigate the robustness and the stability of the solutions as *α* varied, for each responder, we computed the Pearson correlation between regression weights between each pair of *α* values in the set [0.5, 0.6, 0.7, 0.8, 0.9, 0.95, 1]. For each classification task, the average across responders of this correlation was always higher than 0.75.

The regression weights *β* and the bias *β*_*0*_ were estimated minimizing the loss function *L*_*LR*_*(β*, *β*_*0*_*)* via coordinate descent.^44^ The logistic regression models were trained using the Python version (http://hastie.su.domains/glmnet_python) of the Glmnet package.^44^

#### Classifying the choice of the responder

For choice classification, the outcome variable was 0 for rejected offers and 1 for accepted offers. We considered only unfair offers (€1, €2) and fair offers (€4, €5) for this analysis. Mid-range offers (€3) were excluded from this analysis. This analysis was conducted over 18 responders for each direction of movement (leftwards and rightwards). The number of trials available for individual responders is reported in Table S4.

#### Classifying the fairness of the offer

For fairness classification, the outcome variable was 0 for unfair offers (€1, €2) and 1 for fair offers (€4, €5). The €3 offers were excluded from this analysis. The analysis was conducted over 18 responders for each direction of movement (leftwards and rightwards). The number of trials available for individual responders is reported in Table S4.

#### Classifying the choice of the responder using unfair trials only

In this analysis, we classified choice using only the subset of unfair trials. The analysis was conducted over 8 responders for rightward movements and 7 responders for leftward movements. The number of trials available for individual responders is reported in Table S4.

#### Classifying the fairness of the offer using accepted trials only

In this analysis, we classified fairness using only the subset of accepted trials. The analysis was conducted over 8 responders for rightward movements and 7 responders for leftward movements. The number of trials available for individual responders is reported in Table S4.

#### Generalization of responder’s choices to over mid-range offers

In this analysis, the classifiers were trained to classify choice using all but mid-range offers (€3), which were used for testing. This analysis was conducted over 18 responders for each direction of movement (leftwards and rightwards). The number of trials available for individual responders is reported in Table S5.

#### Control analysis using leave-one-subject-out cross-validation

In this control analysis, the logistic regression classifiers were trained to classify choice and fairness using data from all but one responder, and then tested it on the left-out responder. This analysis was conducted over 18 responders for each direction of movement (leftwards and rightwards). The hyperparameter λ was tuned using a nested leave-one-subject-out cross-validation procedure. The best λ value was found within the range logspace(1, −3, 5) using a grid search procedure, and *α* was fixed at 0.95. As a test of the robustness of results, we repeated the analysis with values of α ranging from 0.5 to 1 and using k-fold cross-validation instead of leave-one-subject-out cross-validation. In all cases, prediction performance remained at chance for both choice and fairness.

#### Alternative classification approaches used for comparison

We compared logistic regression (LR in Figure S2 and Table S2) models with a set of alternative models in terms of how well they could predict choice and fairness. The results are shown in (Figure S2 and Table S2). Below, we briefly describe the alternative models used for comparison.

##### Multi-Task LSogistic Regression classifier

Multi-task learning classifiers leverage useful information contained in related tasks to improve classification performance.^45^ Here, we used multi-task learning (MTLR in Figure S2 and Table S2) to simultaneously classify choice (accept versus reject) and fairness (fair versus unfair) from the z-scored multivariate single-trial kinematic vector. The MTLR model was trained by minimizing the negative log likelihood *L*_*MTLR*_ with L2-penalty defined as:

$$\min_{\beta ,\beta_{0}}\;L_{MTLR}\left(\beta ,\beta_{0}\right)=\min_{\beta ,\beta_{0}}\sum_{r=1}^{2}\frac{1}{n}\sum_{i=1}^{n}w_{i,r}\left(\log\left(1+e^{-y_{i,r}\left(\beta_{r}^{T}K_{i,r}+\beta_{0,r}\right)}\right)\right)$$

$$+\lambda_{1}\left(\left(1-\alpha \right)\left\|\beta {\|}_{F}^{2}+\alpha \right\|\beta {\|}_{2,1}\right)+\frac{1-\lambda_{2}}{\lambda_{2}}\sum_{r}^{2}\|\beta_{r}-\frac{1}{2}\sum_{s}^{2}\beta_{s}{\|}^{2}$$

where λ_1_ is the elastic net regularization parameter, λ_2_ is the task-coupling parameter, *r* and *s* are indexes that denote the task (*r,s =1* choice classification, *r,s = 2* fairness classification), *n* is the number of trials, *β*_*t*_ is the regression weight, *β*_*0,r*_ is the bias term, *w*_*i,r*_ is the rescaling weight, *y*_*i,r*_ is the response variable for the given task *r* in the *i-th* trial, and *K*_*i,r*_ is kinematic vector in the *i-th* trial. The hyperparameters λ_1_ and λ_2_ were tuned using a nested LOTO-CV procedure and α was set to 0.95, using a grid search within the range logspace(-3, 3, 7) for λ_1_, and linspace(0.1, 1, 19) for λ_2_. The loss function was minimized using the L-BFGS method.^46^ The MTLR models were implemented using PyTorch.^47^

##### Static Weights Logistic Regression classifier with time-integration

This version of the logistic regression classifier used static (that is, time independent) weights and time integration of evidence, similar to.^43^ Specifically, the Static Weights Logistic Regression (SWLR in Figure S2 and Table S2) model estimated the single-trial cumulative probability P(y(t)|[K(1),…,K(t)]) (i.e., the cumulative evidence) in favor of an accepted offer and a fair offer as a function of the time-dependent kinematic vector in that trial up to time *t*, as follows:

$$P\left(\left[y\left(0\right)=1\right]\right)=P\left(\left[y\left(0\right)=0\right]\right)=\frac{1}{2}$$

$$P\left(\left[y\left(t\right)=0\right]\;|\;K\left(1\right),\ldots ,K\left(t\right)\right)=\sigma \left(\beta_{0}+K\left(t\right)\cdot \beta +w\cdot \left(y\left(t-1\right)-\frac{1}{2}\right)\right)$$

P([y(t)=1]|K(1),…,K(t))=1−P([y(t)=0]|K(1),…,K(t))

where σ is the sigmoid function, is a bias term, *K(t)* is the kinematic vector at time epoch t, *β* is the vector of the regression coefficients (weights), and *w* is a coefficient weighting the cumulation of evidence over time. In each trial, classification was performed integrating evidence until the end of the movement (100% of movement time). The model was trained by minimizing the negative binomial log likelihood with L2-penalty via stochastic gradient descent with adaptive moment estimation (Adam).^48^ The hyperparameter λ, controlling the strength of the L2 regularization, was tuned using a nested 5-fold CV procedure. The best hyperparameter λ was found with a grid search within the range logspace(1, −3, 5).

##### Encoding-Decoding classifier

This classifier was similar to that used in.^49^ The Encoding-Decoding (ED in Figure S2 and Table S2) classifier includes an encoding part, which modeled each kinematic feature as a function of the choice of the responder, the fairness of the proposed offer, and their interaction, and a decoding part, which uses the encoding model to compute (using Bayes' theorem) the posterior probabilities of each responder’s outcome given the kinematic parameter. Classification was then made by choosing the response class with the higher posterior probability. The encoding models were linear regression models fitted with the package scikit-learn.^50^ In building response probabilities from the linear regression model, we assumed that noise was Gaussian and, similar to,^49^ that the kinematic features were conditionally independent given the responder’s choice and the offer’s fairness.

##### Gaussian process regression classifier

We finally classified responses using a Bayesian non-linear regression implemented as a Gaussian process regression (GPR in Figure S2 and Table S2) model.^51^

The kinematic vectors were considered as noisy observations of a 170-dimensional latent function f(K) defined as a Gaussian process:

$$f\left(K\right)\;∼\;GP\left(m\left(K\right),c\left(K,K^{\prime }\right)\right)$$

where $K,K^{\prime }$ are any two vectors in kinematic space. The Gaussian process is specified by the analytical form and hyperparameters of a mean function m(K)(in our case set to zero as kinematic vectors were z-scored for this analysis) and covariance function $c\left(K,K^{\prime }\right)$ that specifies the similarity of values between any two vectors in the kinematic space. As a covariance function $c\left(K,K^{\prime }\right)$, we chose a squared exponential with automatic relevance determination (SE-ARD) defined as:

$$c_{SE-ARD}\left(K,K^{\prime }\right)=\sigma_{f}^{2}\;\exp\left(-\frac{1}{2}\sum_{d=1}^{170}\frac{{\left(K_{\left(d\right)}-{K_{\left(d\right)}}^{\prime }\right)}^{2}}{l_{d}^{2}}\right)$$

where the signal variance $\sigma_{f}^{2}$ and the length-scales $l_{d}$ are hyperparameters, and *d* denotes the feature. Unlike the commonly used squared exponential covariance function, which has a single length-scale for all features, this more complex covariance allows for a different length-scale $l_{d}$ for each kinematic feature.^51^

We used a Gaussian likelihood function with hyperparameter $\sigma_{n}^{2}$ to model the level of the response variability, such that any samples of latent function f and observed response y at location K and any new samples (that is, the samples in the test set not used to train the model) of predicted values $f_{*}$ at the unseen kinematic trials $K_{*}$, has the following expression:

$$\left[\begin{array}{c} y\\ f_{*} \end{array}\right]\;∼\;N\left(\begin{array}{cc} 0\text{,} & \left[\begin{array}{cc} C\left(K,K\right)+\sigma_{n}^{2}I & C\left(K,K_{*}\right)\\ C\left(K_{*},K\right) & C\left(K_{*},K_{*}\right) \end{array}\right] \end{array}\right)$$

By conditioning on a set of observed (training) data points K, we obtained a posterior distribution over function values at any unobserved data point, including those in the test set $K_{*}$. To binarily classify the response in each test set data point, we passed the mean of the predictive function $\bar{f}\left(K_{*}\right)$ through a Heaviside function that returned 0 if $\bar{f}\left(K_{*}\right)$ was less than 0.5, +1 if it was greater than 0.5, and performs a random prediction if it was equal to 0.5.

The model hyperparameters $\theta =\left[\sigma_{f}^{2},l_{d},\sigma_{n}^{2}\right]$ were randomly initialized and optimized during training by minimizing the negative log marginal likelihood (NLML),^52^ defined as:

$$-\log\;p\left(y|K,\theta \right)=\frac{1}{2}y^{T}C_{y}^{-1}y+\frac{1}{2}\log\left|C_{y}\right|+\frac{n}{2}\log\;2\;\pi$$

where $C_{y}=C\left(K,K\right)+\sigma_{n}^{2}I$, and $\left|C_{y}\right|$ is the determinant of the covariance matrix $C_{y}$. The NLML was minimized through the Polak-Ribière conjugate gradient (CG) method,^53^ over a maximum of 1000 iterations. Since the NLML could suffer from local optima,^51^ 10 random restarts of the optimizer were performed and the hyperparameters θ associated with the smaller NLML were selected. The GPR model was fitted using the GPflow.^54^ Performance was evaluated using LOTO-CV.

#### Quantification of classification performance

Classification performance was quantified as balanced classification accuracy. The balanced accuracy is the average of true positive and true negative classification rates. The true positive rate is the fraction of positives that are correctly classified as positives, and the true negative rate is the fraction of negatives that are correctly classified as negatives. Balanced accuracy, because it balances accuracies of positive and negative classification groups by their respective sample size, is useful in datasets like ours in which classification outcomes are unbalanced. To avoid overfitting, we computed balanced accuracies on test data using a LOTO-CV for all classifications.

#### Computation of the statistical significance of classification performance

To test whether classification performance was significantly above chance, we obtained an overall index of task performance by taking the median of the balanced accuracies across all responders. To create a null-hypothesis distribution of median balanced accuracies under the assumption that there is no relationship between the kinematic data and the choice or fairness in the same trials, we trained the logistic regression models on trial-shuffled data with the choice/fairness labels randomly shuffled across trials (100 random shuffles without replacement per responder). We then computed an empirical p value as *p = r/n*_*c*_, where *n*_*c*_ was the number of samples of the null distribution (100 in this case), and *r* was the number of times where an element of the null distribution was greater than or equal to that of the logistic regression models. To verify the ability of nonparametric permutation tests to maintain a low False Positive Rate (FPR) that matched the threshold set for significance across all sample sizes used in the analyses of Figure 3, following,^55^ we repeated 50 times the analyses in Figure 3 using permuted data with null information (rather than from the real data as in Figure 3). We found that using a threshold of p *= 0.05* for significance, the FPR was stable and close to 0.05 in all the analyses (on average, overall all analyses and permutations, FPR was 0.03%). We also obtained an additional, less conservative, random-guess null-hypothesis distribution of 1000 median balanced accuracies using a random classifier with a prior random guess, i.e., a model that generates predictions by respecting the training set’s class distribution. Then, an empirical p value was computed as above.

#### Computation of the statistical significance of the values of individual logistic regression weights

With LOTO-CV, one set of regression weights is obtained for each left-out trial. For each responder, there are as many sets of regression weights as the number of available trials. To obtain a single set of regression weights from these data, we computed the median of the coefficients across all regression models for a given responder, after removing outliers outside the median ± 2.5 times the median absolute deviation. The same procedure (but without removing the outliers) was also applied to regression models trained on trial-shuffled data (100 random shuffles without replacement of the trial labels) to generate a null-hypothesis distribution of the values of weights of features under the assumption that there is no relationship between the kinematic data and the choice or fairness in the same trial. The removal of the outliers only in the data distribution but not in the null-hypothesis distribution ensures an ultra-conservative determination of features with significant weights. We then computed from this distribution a two-tailed empirical p value of the hypothesis that the value of the coefficient (positive or negative) reflected only a random relationship between kinematics and choice or fairness. The p values of the non-zero coefficients were FDR corrected (*α = 0.05*) for multiple comparisons across all non-zero coefficients.

#### Computation of cross-correlations of logistic regression coefficients across participants

For each pair of responders, we quantified the similarity of choice or fairness encoding as the Pearson correlation (across kinematic variables and time epochs) between the median across all LOTO-CV models of the absolute value of the encoding weights of the first responder and the same quantity of the second responder.

#### Computation of the statistical significance of features concentration across participants

For each feature, we quantified *x*, the number of responders who had a significant weight for that feature. If significant weights were distributed randomly across the kinematic feature space, then *x* would follow a null-hypothesis binomial distribution with probability *p = M/(170·n)*, where *M* is the number of significant weights found over the *n* responders. To test the hypothesis that the concentration of responders with significant weights of a feature was higher than expected if significant weights were distributed randomly across the kinematic feature space, we thus used a one-tailed binomial test with parameters *x*, *n*, and *p* listed above.

## Data Availability

• All data needed to evaluate the conclusions in the paper have been deposited at Mendeley Data: https://data.mendeley.com/datasets/jrtjw734r3 and are publicly available as of the date of publication. • The code supporting the main results of this study is based on freely available software packages listed in the key resources table. • Any additional information required to reanalyze the data reported in this paper is available from the lead contact upon request.
