# Supplementary material for: Decoding social decisions from movement kinematics
Source: iScience. 2022 Nov 10;25(12):105550. doi: 10.1016/j.isci.2022.105550 (PMC9700015; doi:10.1016/j.isci.2022.105550)
Supplement: Document S1. Figures S1–S7 and Tables S1–S6 [file mmc1.pdf]

iScience, Volume *25*

Supplemental information

Decoding social decisions from movement kinematics

Giacomo Turri, Andrea Cavallo, Luca Romeo, Massimiliano Pontil, Alan Sanfey, Stefano Panzeri, and Cristina Becchio

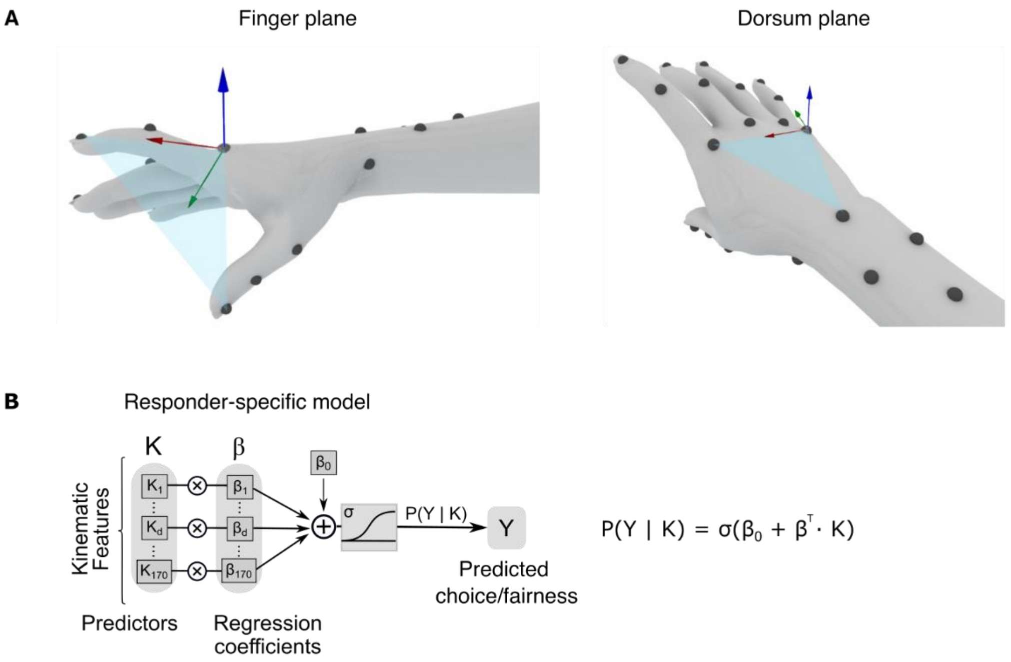

Figure S1. Marker layout and block-diagram of the responder-specific logistic regression model, Related to STAR Methods. (**A**) Marker layout used to reconstruct hand/arm movements. Markers are represented as grey semi spheres. Blu areas indicate the finger plane (left) and dorsum plane (right). The red, green, and blue arrows refer to the *x*-, *y*-, and *z*-coordinates of the finger/dorsum plane. (**B**) Block-diagram and equation of the responder-specific logistic regression model used to estimate choice and fairness information encoded in movement kinematics.

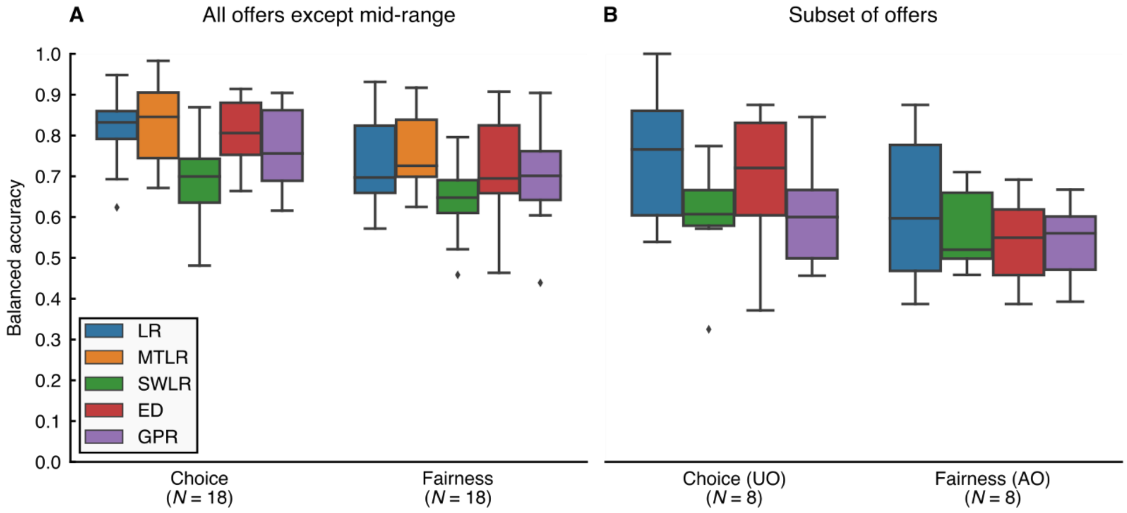

Figure S2. Comparison of logistic regression with alternative classification approaches, Related to STAR Methods. (**A**) Boxplots of balanced prediction accuracies of choice and fairness obtained using Logistic Regression (LR), Multi-Task Logistic Regression (MTLR), Static Weights Logistic Regression (SWLR), Encoding-Decoding (ED), and Gaussian Process Regression (GPR). Classifiers were trained on rightward movements. (**B**) Boxplots of balanced prediction accuracies of choice on unfair trials only (UO), and fairness on accept trials only (AO) obtained using LR, SWLR, ED, and GPR. These analyses corroborate our choice of using the LR classifier for the main analyses of this article, because the LR performed at least as well as any other alternative classifier and was simple to interpret. *N* indicates the number of responders included in each analysis.

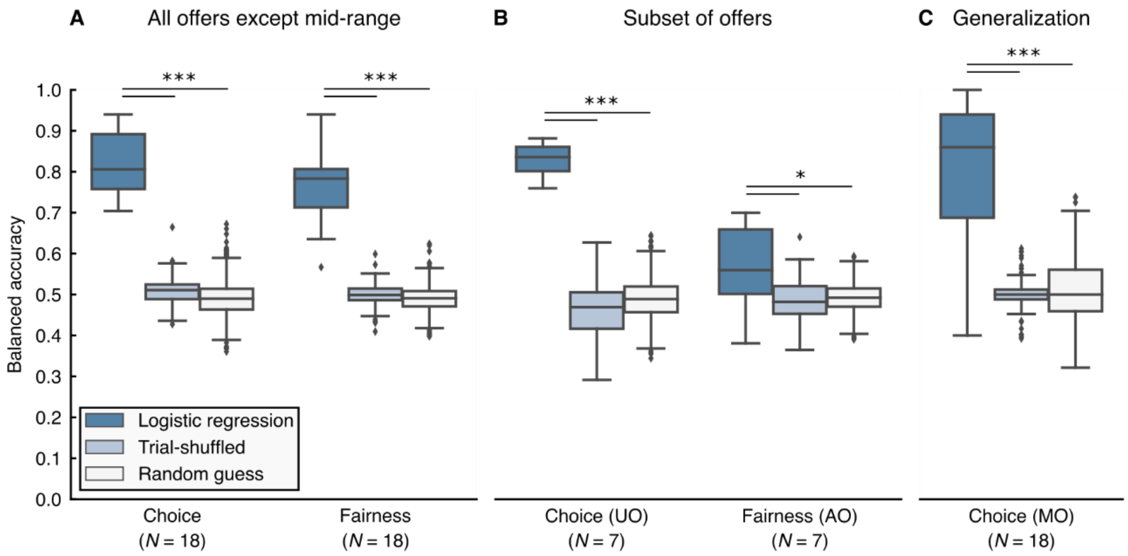

Figure S3. Performance of logistic regression classifiers trained with leftward movements, Related to Figure 3. (**A**) Boxplots of balanced prediction accuracies of responder-specific logistic regression classifiers trained on leftward movements to predict choice and fairness. Prediction accuracies were significantly higher for actual data than trial-shuffled data and random guesses. (**B**) Boxplots of balanced prediction accuracies of choice on unfair trials only (UO) and fairness on accept trials only (AO). (**C**) Boxplot of balanced prediction accuracies of choice on mid-range offers (MO). * indicates $p < 0.05$, ** indicates $p < 0.01$, and *** indicates $p < 0.001$. *N* indicates the number of responders included in each analysis.

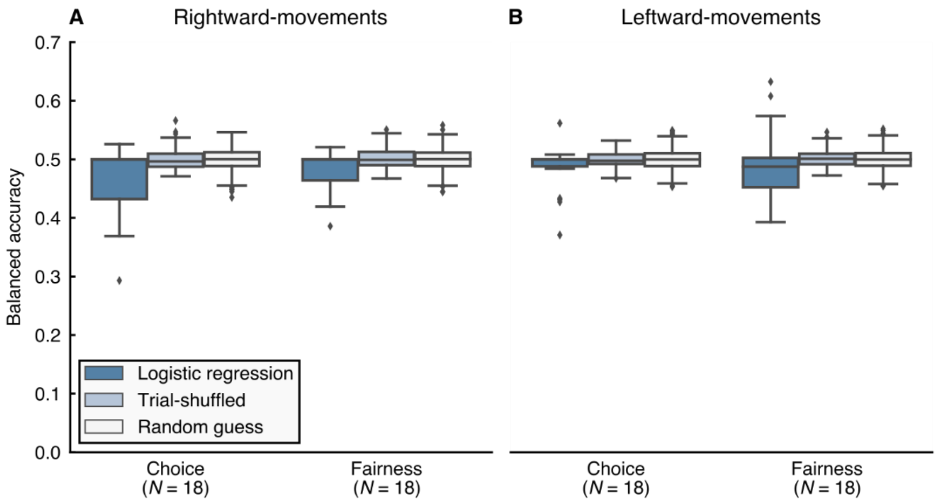

Figure S4. Performance of logistic regression classifiers trained using a leave-one-subject-out testing approach, Related to Figure 3. (**A**) Boxplots of balanced prediction accuracies of leave-one-subject-out (LOSO) logistic regression classifiers trained on rightward movements to predict choice and fairness. (**B**) Boxplots of balanced prediction accuracies of LOSO logistic regression classifiers trained on leftward movements to predict choice and fairness. For both rightward and leftward movements, prediction accuracies of LOSO classifiers trained with actual data were not significantly different from trial-shuffled data and random guesses. *N* indicates the number of responders included in each analysis.

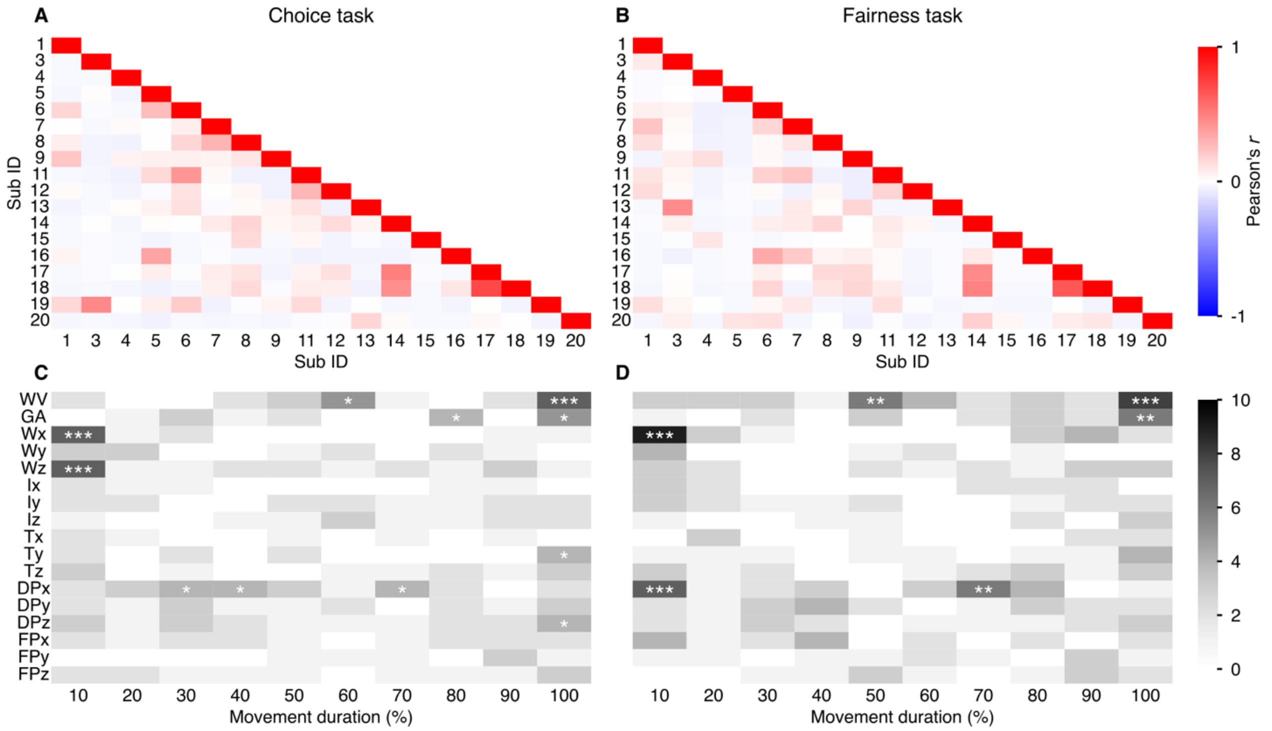

Figure S5. Overlap of choice and fairness weights across responders of leftward movements, Related to Figure 5. (**A**) Pearson correlation of the average logistic regression weights between each pair of responders for leftward movements for classification of choice. (**B**) Pearson correlation of the average logistic regression weights between each pair of responders for leftward movements for classification of fairness. (**C**) Number of responders, for each feature, for which the feature was statistically significant for the classification of choice from leftward movements. (**D**) Number of responders, for each feature, for which the feature was statistically significant for the classification of fairness from leftward movements. * indicates p < 0.05, ** indicates p < 0.01, and *** indicates p < 0.001.

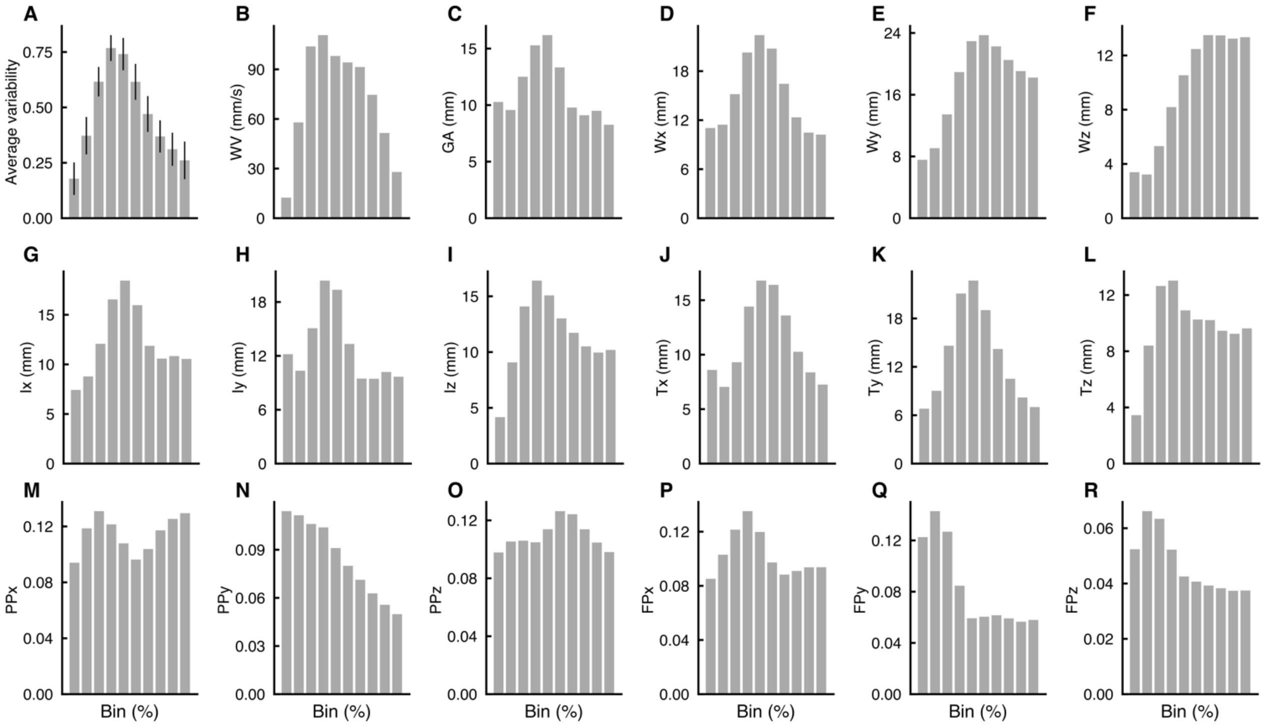

Figure S6. Inter-individual variability of rightward movements, Related to Figure 5. (**A**) Average (± SEM) of the normalized inter-individual variability across kinematic variables for the rightward movements. (**B**-**R**) Inter-individual variability for each kinematic variable for the rightward movements.

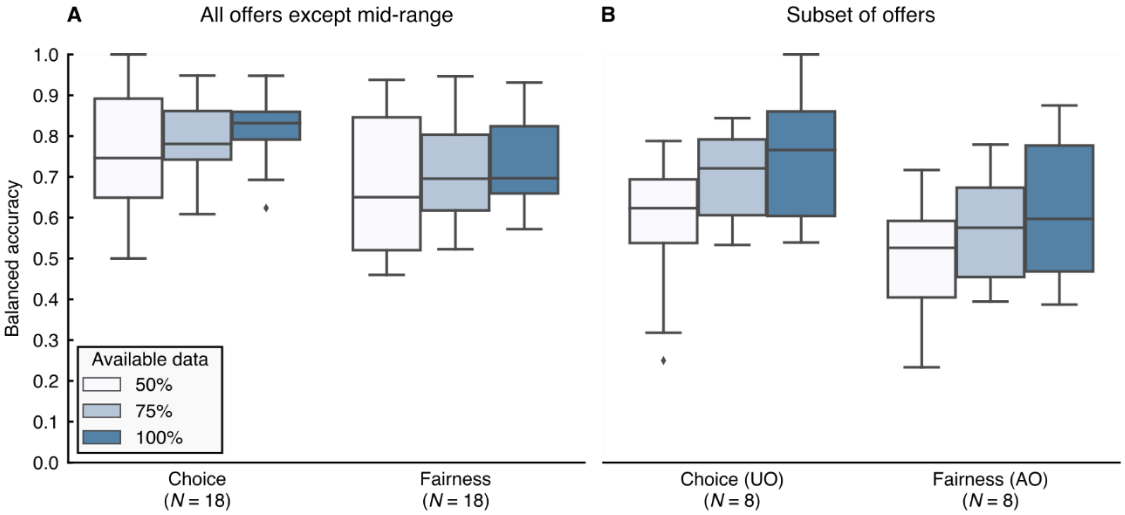

Figure S7. Performance of logistic regression models as a function of the amount of responder-specific data, Related to STAR Methods. (**A**) Boxplots of balanced prediction accuracies of choice and fairness using 50%, 75% and 100% of rightward movements available for each responder. (**B**) Boxplots of balanced prediction accuracies of choice on unfair trials only (UO) and fairness on accept trials only (AO) using 50%, 75% and 100% of the rightward movements available for each responder. *N* indicates the number of responders included in each analysis.

Responder-specific logistic regression models

Rightward movements

				Trial-shuffled			Random guess		
	**Median**	**Q1**	**Q3**	**std**	**z**	***p***	**std**	**z**	***p***
Choice	.832	.791	.860	0.027	11.600	<0.001	0.037	8.758	<0.001
Fairness	.697	.659	.824	0.028	7.986	<0.001	0.037	6.409	<0.001
Choice (unfair only)	.766	.604	.860	0.057	5.072	<0.001	0.032	8.182	<0.001
Fairness (accepted only)	.597	.468	.777	0.044	3.310	<0.001	0.026	4.734	<0.001
Choice (mid-range)	.900	.800	.989	0.047	7.200	<0.001	0.073	3.342	<0.001

Leftward movements

				Trial-shuffled			Random guess		
	**Median**	**Q1**	**Q3**	**std**	**z**	***p***	**std**	**z**	***p***
Choice	.806	.758	.892	0.034	9.070	<0.001	0.043	7.576	<0.001
Fairness	.783	.713	.807	0.029	9.284	<0.001	0.030	9.217	<0.001
Choice (unfair only)	.836	.801	.861	0.066	5.483	<0.001	0.048	7.114	<0.001
Fairness (accepted only)	.559	.501	.659	0.045	1.770	.07	0.032	2.321	<0.05
Choice (mid-range)	.860	.687	.940	0.040	7.485	<0.001	0.074	3.890	<0.001

Leave-one-subject-out logistic regression models

Rightward movements

				Trial-shuffled			Random guess		
	**Median**	**Q1**	**Q3**	**std**	**z**	***p***	**std**	**z**	***p***
Choice	.500	.433	.539	0.015	-0.427	.550	0.020	-0.385	.505
Fairness	.474	.422	.530	0.015	-1.168	.960	0.020	-0.898	.903

Leftward movements

				Trial-shuffled			Random guess		
	**Median**	**Q1**	**Q3**	**std**	**z**	***p***	**std**	**z**	***p***
Choice	.498	.458	.551	0.015	-0.414	.510	0.019	-0.346	.519
Fairness	.488	.443	.529	0.014	-0.797	.820	0.019	-0.471	.719

Table S1. Performance of single-subject and leave-one-subject-out logistic regression models against trial-shuffled data and random guesses, Related to Figure 3. We report the median, Q1, and Q3. All *p*-values are computed by comparing the median value to the null-hypothesis distribution computed on trial-shuffled data and random guesses. For reference only, we also report (without using them to compute the *p*-value) the z-scores of the tested values with respect to the mean and standard deviation (std) of the null-hypothesis distributions.

Comparison of logistic regression with alternative classification approaches

Choice

	df	t	*p* (two-sided)
LR vs MTLR	17	-0.28	.962
LR vs SWLR	17	8.04	<0.001
LR vs ED	17	0.72	.962
LR vs GPR	17	3.30	<0.05

Fairness

	df	t	*p* (two-sided)
LR vs MTLR	17	-1.25	.680
LR vs SWLR	17	4.38	<0.01
LR vs ED	17	0.22	.827
LR vs GPR	17	0.98	.682

Choice (unfair only)

	df	t	*p* (two-sided)
LR vs SWLR	7	3.81	<0.05
LR vs ED	7	1.75	.124
LR vs GPR	7	3.16	<0.05

Fairness (accepted only)

	df	t	*p* (two-sided)
LR vs SWLR	7	0.97	.613
LR vs ED	7	1.45	.573
LR vs GPR	7	1.10	.613

Table S2. Comparison of logistic regression with alternative approaches, Related to STAR Methods. We report degrees of freedom, t statistics, and p-values of two-sided paired t-tests conducted to compare the performance of logistic regression (LR) with alternative models (MTLR, SWLR, ED, and GPR) for choice, fairness, choice (unfair only), and fairness (accepted only) classification of rightward movements. All *p*-values are Holm-Bonferroni corrected for the number of comparisons listed for each entry.

Overlap of choice and fairness weights across responders
Rightward movements

	**Responders (N)**	**Test probability**	***p* (one-sided)**
Choice	5 (18)	246/(170*18)	<0.05
	6 (18)	246/(170*18)	<0.01
	7 (18)	246/(170*18)	<0.001
Fairness	5 (18)	290/(170*18)	<0.05
	6 (18)	290/(170*18)	<0.01
	7 (18)	290/(170*18)	<0.001

Binomial tests for
Leftward movements

	**Responders (N)**	**Test probability**	***p* (one-sided)**
Choice	4 (18)	239/(170*18)	<0.05
	6 (18)	239/(170*18)	<0.01
	7 (18)	239/(170*18)	<0.001
Fairness	5 (18)	272/(170*18)	<0.05
	6 (18)	272/(170*18)	<0.01
	7 (18)	272/(170*18)	<0.001

Table S3. Summary of binomial tests for overlap of choice and fairness weights across responders, Related to Figure 5. We report the number of responders who had a significant weight for a given feature, and in brackets, the total number of responders, the test probability, computed as the number of significant weights over the total number of responders divided by the number of the possible significant weights (number of features * total number of responders), and the *p*-value of the one-sided binomial test.

Number of trials for choice and fairness classification

Rightward movements

ID	Choice			Fairness			Choice (unfair only)			Fairness (accepted only)		
	**R**	**A**	**total**	**UF**	**F**	**total**	**R**	**A**	**total**	**UF**	**F**	**total**
1	12	38	50	19	31	50	12	7	19	7	31	38
2	0	55	55	24	31	55	0	24	24	24	31	55
3	12	38	50	19	31	50	12	7	19	7	31	38
4	25	32	57	25	32	57	25	0	25	0	32	32
5	19	30	49	19	30	49	19	0	19	0	30	30
6	7	35	42	13	29	42	7	6	13	6	29	35
7	12	42	54	24	30	54	12	12	24	12	30	42
8	27	32	59	27	32	59	25	2	27	2	30	32
9	16	29	45	16	29	45	16	0	16	0	29	29
10	1	51	52	21	31	52	1	20	21	20	31	51
11	21	32	53	21	32	53	21	0	21	0	32	32
12	22	32	54	24	30	54	21	3	24	3	29	32
13	29	29	58	27	31	58	23	4	27	4	25	29
14	23	29	52	23	29	52	23	0	23	0	29	29
15	22	31	53	22	31	53	22	0	22	0	31	31
16	18	41	59	26	33	59	17	9	26	9	32	41
17	21	39	60	27	33	60	20	7	27	7	32	39
18	24	32	56	24	32	56	24	0	24	0	32	32
19	26	33	59	25	34	59	24	1	25	1	32	33
20	27	32	59	27	32	59	23	4	27	4	28	32

Leftward movements

ID	Choice			Fairness			Choice (unfair only)			Fairness (accepted only)		
	**R**	**A**	**total**	**UF**	**F**	**total**	**R**	**A**	**total**	**UF**	**F**	**total**
1	17	43	60	28	32	60	16	12	28	12	31	43
2	0	56	56	24	32	56	0	24	24	24	32	56
3	16	45	61	29	32	61	16	13	29	13	32	45
4	24	30	54	24	30	54	24	0	24	0	30	30
5	23	38	61	28	33	61	22	6	28	6	32	38
6	16	50	66	34	32	66	16	18	34	18	32	50
7	12	45	57	25	32	57	12	13	25	13	32	45
8	21	30	51	20	31	51	20	0	20	0	30	30
9	24	40	64	32	32	64	23	9	32	9	31	40
10	2	55	57	25	32	57	2	23	25	23	32	55
11	24	31	55	25	30	55	24	1	25	1	30	31
12	23	30	53	22	31	53	21	1	22	1	29	30
13	27	28	55	22	33	55	20	2	22	2	26	28
14	25	33	58	25	33	58	24	1	25	1	32	33
15	23	32	55	23	32	55	22	1	23	1	31	32
16	15	37	52	22	30	52	15	7	22	7	30	37
17	18	31	49	19	30	49	18	1	19	1	30	31
18	24	32	56	24	32	56	24	0	24	0	32	32
19	23	30	53	23	30	53	23	0	23	0	30	30
20	22	28	50	21	29	50	20	1	21	1	27	28

Table S4. Number of trials for individual responders, Related to STAR Methods. For each responder (ID), we report the number of trials available for choice and fairness classification and the distribution among classes (R=rejected, A=accepted, UF=unfair, F=fair).

Number of trials for choice classification of mid-range offers

ID	*Rightward movements*						*Leftward movements*					
	**Training set**			**Test set (3€ offers)**			**Training set**			**Test set (3€ offers)**		
	**R**	**A**	**total**	**R**	**A**	**total**	**R**	**A**	**total**	**R**	**A**	**total**
1	12	38	50	0	8	8	17	43	60	4	10	14
2	0	55	55	0	11	11	0	56	56	0	11	11
3	12	38	50	0	10	10	16	45	61	1	11	12
4	25	32	57	4	10	14	24	30	54	2	7	9
5	19	30	49	0	11	11	23	38	61	1	11	12
6	7	35	42	0	12	12	16	50	66	0	11	11
7	12	42	54	0	10	10	12	45	57	1	11	12
8	27	32	59	0	1	1	21	30	51	11	11	22
9	16	29	45	0	1	1	24	40	64	10	11	21
10	1	51	52	0	12	12	2	55	57	0	11	11
11	21	32	53	9	1	10	24	31	55	10	1	11
12	22	32	54	10	7	17	23	30	53	5	1	6
13	29	29	58	4	6	10	27	28	55	6	7	13
14	23	29	52	10	0	10	25	33	58	12	0	12
15	22	31	53	7	2	9	23	32	55	10	3	13
16	18	41	59	2	11	13	15	37	52	0	9	9
17	21	39	60	4	11	15	18	31	49	0	8	8
18	24	32	56	12	0	12	24	32	56	10	0	10
19	26	33	59	0	11	11	23	30	53	0	12	12
20	27	32	59	8	6	14	22	28	50	4	4	8

Table S5. Number of trials for choice classification of mid-range offers for individual responders, Related STAR Methods. For each responder (ID), we report the number of trials available for the training and test set (R=rejected, A=accepted).

Random effects structure selection (Fixed effects: Gender × Proposer Gender)

**Model**	Random effects	BIC	Deviance	
m_0	Subject (intercept and Proposer Gender slope)	3364.4	3308.9	(singular fit)
**m_1**	**Subject (intercept)**	**3348.5**	**3308.9**	
m_2	null	3599.2	3567.5	

Fixed effects structure selection (Random effects: Subject intercept)

**Model**	Fixed effects	BIC	Deviance	LRT
m_1	Responder Gender × Proposer Gender	3348.5	3308.9	
m_2	Responder Gender + Proposer Gender	3341.5	3309.8	vs. m_1: $p > .05$
m_3	Responder Gender	3333.8	3310.0	vs. m_3: $p > .05$
m_4	Proposer Gender	3333.6	3309.9	vs. m_3: $p > .05$
**m_5**	**null**	**3325.9**	**3310.0**	**vs. m_4, m_5: $p > .05$**

Table S6. Mixed Effects Model selection, Related to STAR Methods. We used Logistic Mixed Effects Models Effects to assess the effect of *Responder Gender* and *Proposer Gender (silhouette)*. The notation *Gender × Proposer Gender* indicates both main effects of *Responder Gender* and *Proposer Gender*, and their interaction were included in the model. Retained models are highlighted in bold.
